# Preparation, Characterization, and In Vivo Pharmacokinetic Study of the Supercritical Fluid-Processed Liposomal Amphotericin B

**DOI:** 10.3390/pharmaceutics11110589

**Published:** 2019-11-08

**Authors:** Chang-baek Lim, Sharif Md Abuzar, Pankaj Ranjan Karn, Wonkyung Cho, Hee Jun Park, Cheong-Weon Cho, Sung-Joo Hwang

**Affiliations:** 1 College of Pharmacy, Chungnam National University, Daejeon 34134, Korea; syc0745@naver.com; 2 College of Pharmacy, Yonsei University, Incheon 21983, Korea; sumonzar@gmail.com (S.M.A.); pankajkarna@hotmail.com (P.R.K.); dlit83@naver.com (W.C.); 3 Yonsei Institute of Pharmaceutical Sciences, Yonsei University, Incheon 21983, Korea; 4 Department of Industrial and Physical Pharmacy, College of Pharmacy, Purdue University, 575 Stadium Mall Drive, West Lafayette, IN 47907, USA

**Keywords:** Amphotericin B, liposomes, supercritical fluid, hemolysis, AmBisome^®^, pharmacokinetic

## Abstract

Here, we aimed to prepare and optimize liposomal amphotericin B (AmB) while using the supercritical fluid of carbon dioxide (SCF-CO_2_) method and investigate the characteristics and pharmacokinetics of the SCF-CO_2_-processed liposomal AmB. Liposomes containing phospholipids, ascorbic acid (vit C), and cholesterol were prepared by the SCF-CO_2_ method at an optimized pressure and temperature; conventional liposomes were also prepared using the thin film hydration method and then compared with the SCF-CO_2_-processed-liposomes. The optimized formulation was evaluated by in vitro hemolysis tests on rat erythrocytes and in vivo pharmacokinetics after intravenous administration to Sprague-Dawley rats and compared with a marketed AmB micellar formulation, Fungizone^®^, and a liposomal formulation, AmBisome^®^. The results of the characterization studies demonstrated that the SCF-CO_2_-processed-liposomes were spherical particles with an average particle size of 137 nm (after homogenization) and drug encapsulation efficiency (EE) was about 90%. After freeze-drying, mean particle size, EE, and zeta potential were not significantly changed. The stability study of the liposomes showed that liposomal AmB that was prepared by the SCF method was stable over time. In vivo pharmacokinetics revealed that the SCF-CO_2_-processed-liposomes were bioequivalent to AmBisome^®^; the hemolytic test depicted less hematotoxicity than Fungizone^®^. Therefore, this method could serve as a potential alternative for preparing liposomal AmB for industrial applications.

## 1. Introduction

Amphotericin B (AmB), a polyene antibiotic that is produced from *Streptomyces nodosus*, is widely used against life-threatening systemic infections by fungi, such as *Candida albicans* or *Aspergillus fumigates,* as well as parasites, such as *Leishmania donovani* [1,2]. AmB has been clinically used since the early 1960s due to its extremely low rate of resistance (specifically against systemic candidiasis) [3]. Moreover, the broadest antifungal spectrum of AmB ensures its preeminence over other antifungal agents for the treatment of systemic fungal infections and visceral leishmaniasis for more than 30 years [4,5].

AmB has a very low solubility (about 1 mg/L in water) and low membrane permeability; therefore, it is categorized in class IV in the Biopharmaceutical Classification System [6]. The low solubility of AmB is believed to be attributed to the self-association of AmB molecules at a concentration of approximately 0.2 µg/mL, which below the critical micellar concentration (3 µg/mL) [7]. A conventional dosage form of AmB, Fungizone^®^ (Apothecon^®^, Princeton, NJ, USA), a colloidal dispersion with detergents (sodium deoxycholate and buffer), and being available as an injectable, was supposed to resolve these solubility issues; however, hemolysis and dose-dependent nephrotoxicity were reported upon prolonged administration [8,9].

For over two decades, research has been directed toward incorporating AmB into lipid-based drug delivery systems to reduce its toxicity and increase its therapeutic index. Lipid-based systems are able to retain AmB much better than surfactant micelles; thus, these delivery systems release the drug slowly in the form of monomers, reducing its toxicity to mammalian cells. Based on pioneering work in the 1980s [10], a large number of systems have been developed, and three of them, AmBisome^®^, Amphotec^®^, and Abelcet^®^, are now commercially available [11].

AmBisome^®^ (Gilead Sciences Inc, San Dimas, CA, USA) is the only true liposomal formulation of the three, with small unilamellar phospholipid vesicles of less than 100 nm [12]. This formulation is composed of hydrogenated soy phosphatidylcholine (HSPC), cholesterol, distearoylphosphatidylglycerol (DSPG), and AmB in a molar ratio of 2:1:0.8:0.4 [13]. The nano-size of the liposomes ensures that they have a long circulating half-life and good penetration into the tissues. The stable bilayer composition reduces the exchanges with lipoproteins and contributes to the very low toxicity of this formulation [14]. Amphotec^®^ (Three Rivers Pharmaceuticals, LLC, Cranberry Township, PA, USA) is a lyophilized powder that is composed of complexes between cholesteryl sulfate and AmB in a 1:1 equimolar proportion. This formulation, upon reconstitution, forms a colloidal dispersion of microscopic unique disc-shaped particles of uniform size (120 nm in diameter × 4 nm thick) [15]. Abelcet^®^ (Enzon Pharmaceuticals, Inc., Bridgewater, NJ, USA), a lipid complex, is formulated by complexing AmB with synthetic phospholipids (dimyristoylphosphatidylcholine (DMPC) and dimyristoylphosphatidylglycerol (DMPG)) in a 1:1 molar ratio. This formulation possesses a ribbon-like structure, 1 to 10 micrometers in length, and, due to its large size, Abelcet^®^ is rapidly cleared from circulation via the reticuloendothelial system [1,16].

These commercial lipid-based formulations reduce the toxicity of AmB to varying degrees and exhibit different pharmacokinetic profiles [17]; moreover, they considerably improve the therapeutic index of the drug as compared to Fungizone^®^. Among these formulations, the liposomal formulation (AmBisome^®^) has significantly lower toxicity when compared to the other formulations, thus permitting larger doses to be administered; accordingly, AmBisome^®^ is more effective than the other formulations [18]. However, despite the advantages of the liposomal formulation, these preparations are not easily accessible because of the high cost of manufacturing and the preparation method used, which is rather complex for scaling-up [19]. Therefore, an alternate simple, scalable, and cost-effective liposomal preparation method is needed for rectifying these issues. 

The supercritical fluid of carbon dioxide (SCF-CO_2_) is an effective alternative to the traditional method of liposomal preparation as the solvent properties of the supercritical fluid (SCF) can be easily adjusted by altering the process parameters (temperature and pressure) [20]. Recently, this technology has emerged as one of the major techniques for the preparation of liposomes [21]. In our previous studies with liposomal cyclosporine A, we were able to prove the advantages of this novel SCF-CO_2_ method over the conventional Bangham film method [22,23]; in our method, cost-effective liposomes were produced with improved efficacy and stability. Liposomal AmB was investigated in the present study to strengthen and broaden the applications of this newly developed SCF-CO_2_ method. However, an earlier study [24] had reported that the preparation process of gas antisolvent (GAS)-assisted SCF-CO_2_-mediated liposomal AmB was rather complex and it produced microsized liposomes. Thus, in the present study, we used a different approach, i.e., the supercritical antisolvent (SAS) process; here, the phospholipids were coated onto the surface of lactose particles, forming a thin film, which, on hydration, yielded nanosized multilamellar AmB liposomes.

Thus, overall, this study aimed to develop an alternative liposomal formulation for AmB while using the simplest SCF-CO_2_ method and examine the effects of various factors, i.e., organic solvents, temperature and pressure of the process, lipid composition, microfluidization, and lyophilization on liposomal AmB properties (particle size, polydispersity index (PDI), encapsulation efficiency (EE), yield, morphology, etc.). The in vivo pharmacokinetics were assessed, and hemolytic tests were performed and compared with the reference formulations, Fungizone^®^ and AmBisome^®^, to confirm the in vivo efficacy of the prepared liposomes.

## 2. Materials and Methods

### 2.1. Materials

AmB was procured from North China Pharmaceutical Huasheng Co. Ltd. (Hebei, China). Hydrogenated soy phosphatidylcholine (HSPC), distearoylphosphatidylglycerol (DSPG), cholesterol, and hydrochloric acid (37%) were purchased from Sigma-Aldrich (St. Louis, MO, USA). Hanmi Gas Co. Ltd. supplied carbon dioxide with a high purity of 99.9% (Seoul, Republic of Korea). Ethyl alcohol (purity 99.9%), methyl alcohol (MeOH, purity 99.5%), chloroform (CHCl_3_, purity 99.5%), dimethyl sulfoxide (DMSO, purity 99.5%), *N*,*N*-dimethylacetamide (DMA, purity 99.5%), and *N*,*N*-dimethylformamide (DMF, purity 99.5%) were purchased from Samchun Pure Chemical (Pyeongtaek-si, Republic of Korea). All other chemicals and reagents were of analytical grade. Purified water of Milli-Q quality (Milli-Q Reference, Millipore^®^, Molshiem, France) was used throughout the study.

### 2.2. High-Performance Liquid Chromatography of AmB 

The AmB content in the liposome formulations was determined by high-performance liquid chromatography (HPLC) [25]. Liposomes were ruptured by MeOH and CHCl_3_ (1:1) and the solution was then diluted 10-fold with MeOH. The liposomes were filtered through polytetrafluoroethylene (PTFE) syringe filters with 0.45-μm pore size Whatman^®^ syringe filters. The chromatographic analyses were performed on a HPLC Agilent 1200 Infinity Series HPLC system (Agilent Technologies, Waldbronn, Germany), using a C_18_ analytical column (Waters; 250 × 4.6 mm, 5 µm). The mobile phase consisted of (A) MeOH containing 0.1% *v/v* trifluoroaceticacid and (B) water containing 0.1% *v/v* trifluoroaceticacid, with a gradient flow of 60–84% (A) over 9 min. The flow rate was 1 mL/min. (Model 1260 Quat Pump VL), sample injection volumes were 20 µL (Model 1260 ALS), and the detection wavelength was 390 nm.

### 2.3. Solubility and Stability of AmB in Organic Solvents 

The solubility of AmB was determined in DMSO, DMA, DMF, and an equivolume solution of MeOH and CHCl_3_. In addition, these solvents were acidified while using ascorbic acid (vit C) and HCl and the solubility of AmB was measured. The organic solvents were acidified in the following two ways: first, 200 mg vit C was dissolved in 2 mL of DMSO, DMA, and DMF, respectively (vit C was not soluble in an equivolume solution of MeOH and CHCl_3_) and second, 75 μL of 2.5 M HCl was slowly added to 2 mL of DMSO, DMA, DMF, and an equivolume solution of MeOH and CHCl_3_, respectively. An excess (approximately 500 mg) of AmB was placed in a capped glass tube containing 2 mL of the respective organic solvent or acidified organic solvent. The samples were then shaken (60 rpm) at 65 °C in a shaking water bath. After 2 h, the samples were filtered through a 0.45 μm-pore sized PTFE syringe filter (Whatman^®^, Pittsburgh, PA, USA). The filtrate was then diluted while using DMSO and/or MeOH; finally, the AmB concentration in the filtrate was determined using HPLC.

Similarly, the chemical stability of AmB was determined in DMSO and other acidified organic solvents with a relatively high solubility. Five milliliters of AmB solution (10 mg/mL) was transferred to a tube that was incubated at 65 °C in a water bath. Hundred microliter samples of the solution were withdrawn at 0, 5, 15, 30, and 60 min. The samples were then diluted and the content of AmB was analyzed using HPLC.

### 2.4. Preparation of Liposomal AmB by the SCF-CO_2_ Method

**Figure 1:**
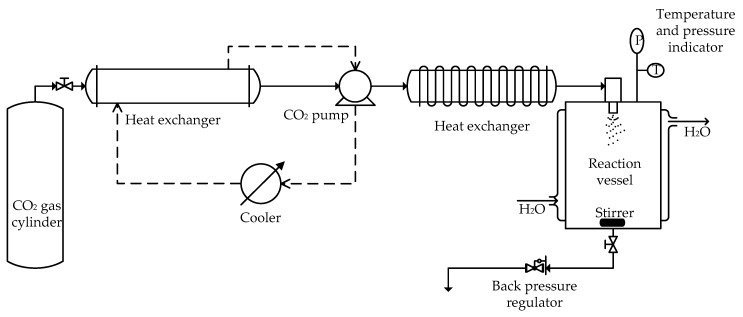
Schematic representation of the experimental apparatus for liposome preparation by the SCF-CO_2_ method.

Liposomes containing AmB were prepared by the SCF-CO_2_ method that was reported in a Korean patent [26]. The experimental apparatus, as shown in Figure 1, was made up of the following components: CO_2_ syringe pump, circulator and cooling lines for maintaining the CO_2_ pump head, and CO_2_, which flowed out of a storage tank (−7 °C); and, a reaction vessel (72 cm^3^) containing a magnetic stirrer, pressure indicator, and temperature indicator. 

For the preparation of liposomes, 84 mg of DSPG was dissolved in 1.0 mL of an equivolume solution of MeOH and CHCl_3_ at 65 °C. Two hundred milligrams of vit C was sonicated to dissolve in 2.0 mL of DMA; next, 50.0 mg of AmB was added to the DMA-vit C solution at 65 °C. The mixture was then transferred to the DSPG solution. The AmB-DSPG lipophilic complex was formed by heating at 65 °C for several minutes. Further, 213 mg of HSPC was dissolved in 1.0 mL of an equivolume solution of MeOH and CHCl_3_ at 65 °C to yield a clear solution. Fifty-two milligrams of cholesterol was also dissolved in a 1.0 mL equivolume solution of MeOH and CHCl_3_ at 65 °C. The cholesterol and HSPC solutions were then mixed with the AmB-DSPG complex solution. The resulting solution and 900 mg (9% *w/v*) of anhydrous lactose were sealed in the reaction vessel. The temperature of the vessel varied between 35–65 °C and the pressure varied between 10–30 MPa. Supercritical CO_2_ was introduced into the vessel until the desired pressure was reached. After approximately 30 min. with stirring at equilibrium, additional supercritical CO_2_ continued to flow into the vessel (with controlled back pressure regulation to maintain the operational pressure) for about 30 min. to wash out any remaining solvent. The vessel was then depressurized to atmospheric pressure; the AmB-phospholipid mixture was coated onto the surface of the lactose particles, forming a thin film. The resulting thin film was then hydrated with 10 mL of Milli-Q water at a temperature of 65 °C to form a liposomal AmB suspension. Liposomes that were obtained using this process were termed SCF-CO_2_ liposomes.

### 2.5. Preparation of Liposomal AmB by the Conventional Method 

The liposomal AmB was also prepared by the conventional thin film hydration method for comparison with the liposomes that were prepared by the SCF-CO_2_ method. AmB was dissolved in an organic solvent and mixed with a solution of phospholipid dissolved in organic solvents in a manner that was similar to that in the SCF-CO_2_ method. The mixture was then transferred to a round-bottom flask and connected to an EYELA rotary evaporator (N-1110V-W; EYELA, Shanghai, China) and water bath (SB-1200; EYELA), with the temperature being maintained at 45 °C with proper mixing. The organic solvents were then removed under reduced pressure to obtain a thin film on the wall of the vessel; the resulting film was hydrated with 9% lactose aqueous solution at a specific temperature of 65 °C. After hydration, multilamellar liposomal AmB was obtained, and the resulting liposomes were sonicated (Beckman XL-80 ultracentrifuge, Branson, MO, USA) for 30 min. for vesicle size reduction.

### 2.6. Lyophilization of Liposomal AmB

Next, the effect of lyophilization of liposomal AmB was evaluated. Samples from Section 2.5. and 2.6. were placed in 50-mL Eppendorf^®^ tubes and freeze-dried for 24 h while using an ilShin BioBase (Seoul, Republic of Korea) freeze-dryer under vacuum (5 mTorr). The samples were pre-frozen at −75 ± 1.0 °C for 24 h prior to final drying at −88 ± 1.0 °C for 24 h. At the end of the lyophilization process, the liposomal AmB was converted into a lyophilized porous cake and the cake was rehydrated with normal saline.

### 2.7. Characterization of Liposomal AmB

#### 2.7.1. Determination of Particle Size, PDI, and Zeta Potential of Liposomal AmB 

The average size, PDI, and zeta potential of liposomes were determined by dynamic light scattering (DLS) while using an electrophoretic light scattering spectrophotometer (ELS-8000, Otsuka Electronics, Osaka, Japan) at a fixed angle of 90° and at room temperature. The system was used in the auto-measuring mode. The DLS analysis was based on the cumulants method. The PDI was also estimated from the correlation function profile using histogram analysis. 

#### 2.7.2. Encapsulation Efficiency Measurement 

The aliquots of liposomes were diluted with distilled water. The resulting liposomal suspensions were then placed in polycarbonate centrifuge tubes (Amicon^®^ Ultra-4) and centrifuged (Beckman XL-80 ultracentrifuge) at 45,000× g and 4 °C for 40 min. to separate the liposomal vesicles from the aqueous solution containing free AmB. The precipitate was dissolved in DMSO-MeOH (1:1) and mixed; the resulting solution was analyzed by HPLC to determine the content of AmB that was encapsulated in the liposomes (Equation 1).

EE % = AmB entrapped in the liposome/AmB in liposomal suspension × 100,(1)

where, AmB entrapped in the liposome and AmB in liposomal suspension are the concentration of AmB in the precipitated liposomal vesicles and total AmB (free and entrapped), respectively.

#### 2.7.3. Small X-ray Scattering

The small X-ray scattering (SASX) pattern was obtained by fine-focused Cu Kα radiation (18 kV) generated by a Rigaku D/MAX-2500 X-ray diffractometer (Rigako Co., Tokyo, Japan). The scattering angle of the diffractogram was in the range of 2θ = 0.165–6° and the radiation wavelength was 1.542 Å. The SAXS images were detected while using an image plate system and the scattering intensity was plotted as a function of the scattering vector q, defined as 4π × sinθ/λ; where, λ and θ are the wavelength and scattering angle, respectively. Experiments were carried out in duplicate to check their reproducibility. The q value and intensity of the peaks were determined through each cycle.

#### 2.7.4. Freeze-Fracture Electron Microscopy

Morphological analysis of liposomes was performed by freeze-fracture electron microscopy. The investigations were performed while using a field emission-scanning electron microscope (FE-SEM; Hitachi S-4700, Tokyo, Japan), operating at 5 kV, and in ultra-high-resolution mode. For freeze-fracture electron microscopy measurements, the liposomes were immediately frozen while using a propane jet-freeze device JFD 030 (BAL-TEC, Balzers, Liechtenstein). Subsequently, the samples were transferred to freeze-fracture/etching/coating system MED 020 GBE (BAL-TEC) using a high vacuum cryo-transfer system VTC-100 (BAL-TEC). The samples were kept below −120 °C in a protected atmosphere during the examination. The frozen samples were fractured using a metal knife, etched for 1 min. at −105 °C and coated with platinum for 70 s at 70 mA. Finally, the samples were observed while using a cryogenic FE-SEM (Hitachi S-4700) at −120 °C. 

#### 2.7.5. Transmission Electron Microscopy

Transmission electron microscopy (TEM) was used to study the morphology of liposomes in a liquid state. TEM images were obtained by a Philips Tecnai G220 S-TWIN microscope (Koninklijke Philips Electronics NV, Amsterdam, Netherland) at an accelerating voltage of 200 kV. The samples were air-dried on a copper grid with carbon coating and the images were observed.

### 2.8. Stability Study 

Liposomal AmB that was prepared by the SCF-CO_2_ and conventional methods was passed through the micro-fluidization process; the resultant nanosized liposomes were used in the stability test. Liposomes were stored at −5 °C. The changes of encapsulated drug content and mean diameter were investigated for 0, 7, 14, 21, and 28 days. The change in the encapsulated drug content was determined using HPLC and the mean diameter was measured with a dynamic light scattering (DLS) apparatus.

### 2.9. Hemolysis Test from Rat Erythrocytes (RBCs) 

After intravenous administration of AmB formulations, the interaction of AmB with red blood cells (RBC) causes their lysis, leading to anemia. Thus, the safety of novel AmB delivery strategies, such as liposomes, can be studied by comparing their hemolysis potential. Venous blood was collected from healthy Sprague-Dawley rats while using cardiac puncture. The whole blood was centrifuged (2000 rpm) for two min., and the supernatant and buffy coat were pipetted off and discarded. The RBCs were diluted using isotonic PBS, pH 7.4, generating a proper absorbance at 576 nm in the presence of 25 μg/mL of Fungizone^®^. To study hemolysis, solutions of RBCs with different levels of AmB were incubated in a shaking water bath (80 rpm) at 37 °C for 30 min. The un-lyzed RBCs were removed by centrifugation (5000 rpm) for 10 min. The supernatant was collected and analyzed for hemoglobin by UV/VIS spectroscopy at a wavelength of 576 nm. The percent of hemolyzed RBCs was determined while using the following equation (Equation 2):

Hemolysis % = (Abs − Abs_0_)/(Abs_100_ − Abs_0_) × 100, (2)

where, Abs, ${\mathrm{Abs}}_{0},$ and ${\mathrm{Abs}}_{100}$ are the absorbance for the sample, in parallel with a control with no AmB and a control in the presence of 25 μg/mL AmB as Fungizone^®^, respectively.

### 2.10. In Vivo Study

#### 2.10.1. Pharmacokinetic Study in Sprague-Dawley Rats 

Male Sprague-Dawley rats were provided by Samtako (Osan, Republic of Korea) for the evaluation of the pharmacokinetic profiles of liposomal AmB. An adaptation period of approximately one week was allowed prior to the experiment with housing in cages and provision of free access to tap water and a pelleted diet. The animal experiment laboratory was maintained under controlled conditions of temperature (25 ± 2 °C), relative humidity (55 ± 10%), and a 12 h/12 h light-dark cycle. The average body weight of the experimental rats was approximately 200 g. The standard deviations for individual body weight within the experiment were ±10 g. All of the experiments were approved by the Institutional Animal Care and Use Committee (IACUC-201910-965-02, 09 January 2019) at Yonsei University, Seoul, Korea, and performed according to the IACUC guidelines. Two kinds of AmB commercial products (AmBisome^®^, Fungizone^®^) and SCF liposomal AmB were administrated as a single dose of 3 mg/kg. A total of 12 rats were randomized into three groups (four rats in each) and treated by tail vein injection as follows; Group 1: AmBisome^®^, Group 2: Fungizone^®^, and Group 3: SCF liposomal AmB. Blood samples were collected from the retro-orbital sinus of the eye into EDTA-treated Eppendorf^®^ tubes. The blood sampling times were 15 min. and 30 min. and 1, 3, 6, 10, and 24 h. The samples were centrifuged at 10,000 rpm (9425 × *g*) for 10 min. at 4 °C. The supernatant plasma was obtained and stored at −80 °C until analysis.

#### 2.10.2. Determination of Plasma AmB

The frozen plasma samples were thawed and 300 μL of plasma was deproteinized by 700 μL of MeOH with 50 μg/mL of 1-amino-4-nitronaphthalene as an internal standard. The samples were vortexed for 5 min. and centrifuged for 15 min. at 17,000 × *g* at 4 °C to precipitate the erythrocytes. The deproteinized supernatant was recovered and 20 μL was injected into the HPLC system to determine the plasma AmB concentration. 

### 2.11. Statistical Analysis

The unpaired Student’s *t*-text was applied to perform statistical analysis. Data are presented as mean ± standard deviations and the significance level was set at *p* < 0.05.

## 3. Results

### 3.1. Screening of Organic Solvent Systems 

**Figure 2:**
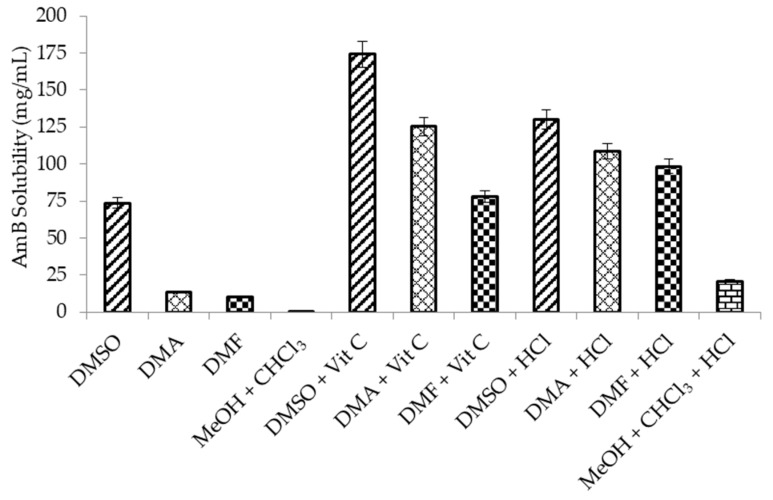
The solubility of amphotericin B (AmB) in different organic solvents at 65 °C. dimethyl sulfoxide (DMSO), *N*,*N*-dimethylacetamide (DMA), *N*,*N*-dimethylformamide (DMF), and an equivolume solution of MeOH + CHCl_3_.

Figure 2 shows the solubility of AmB in organic solvents with or without acidification. The drug solubility was considerably enhanced by acidification of the organic solvents while using vit C and HCl. AmB exhibited high solubility in DMSO, despite the fact that it was not acidified by vit C or HCl. In contrast to DMSO, the drug was poorly soluble in the equivolume solution of MeOH and CHCl_3_. However, when the equivolume solution of MeOH and CHCl_3_ was acidified by HCl, AmB was soluble in the mixture. Vit C was not soluble in an equivolume solution of MeOH and CHCl_3_. Therefore, the solubility experiment was not carried out on the vit C-acidified equivolume solution of MeOH and CHCl_3_. The acidified organic solvents (DMA and DMF) showed solubilities that were comparable to DMSO or even acidified DMSO. 

**Figure 3:**
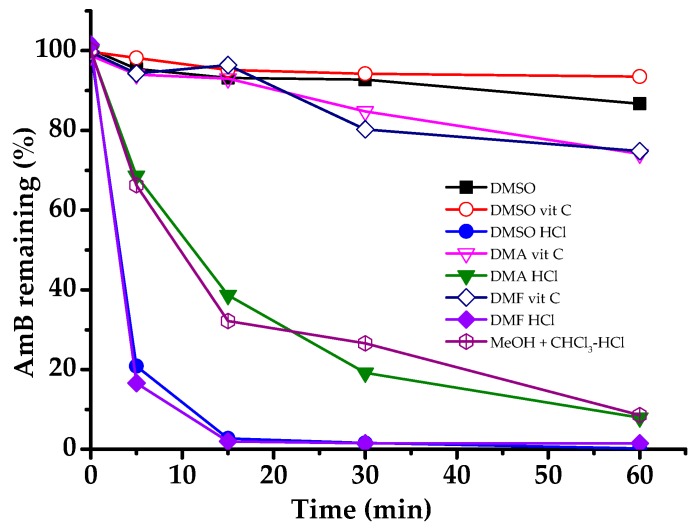
AmB remaining (%) in different organic solvent systems.

The stability of AmB and the effects of vit C and HCl on its stability were evaluated. First, the stability test of AmB was performed in DMSO and acidified organic solvents with relatively high solubility. Figure 3 shows that the remaining percentage of AmB was decreased in different organic solvent systems with different degradation kinetics.

The rapid degradation of AmB was particularly observed in organic solvents that were acidified with HCl. The semi-logarithmic plots of the AmB remaining (in %) against time in different organic solvent systems indicated pseudo-first-order degradation behavior (Equation 3)

log[C]*_t_* = −[(*k*_obs_/2.303) × *t*] + log[C_0_], (3)

where C_0_ is the initial concentration of AmB and C_t_ is the percentage remaining at time *t*, which allowed for the calculation of the degradation pseudo-first-order rate constants (*k*_obs_) as the slopes of the lines, obtained by linear regression analysis. The half-life (*t*_1/2_) values were calculated using Equation 4. Table 1 shows the values of *k*_obs_ and *t*_1/2_.

*t*_1/2_ = 0.693/*k*_obs_, (4)

### 3.2. Optimization of the Preparation Process for Liposomal AmB 

#### 3.2.1. Effect of Organic Solvents on the Preparation of Liposomal AmB 

Table 2 shows the effect of four different solvents on the mean diameter, PDI, and EE % of liposomal AmB prepared while using the SCF-CO_2_ process at constant conditions (45 °C, 20 MPa). The percent EE of liposomal AmB obtained with DMSO-vit C was relatively lower than that obtained using the DMA-vit C and DMF-vit C solvent systems.

The size of liposomal AmB prepared using the MeOH + CHCl_3_-HCl solvent system was the smallest among all of the samples. However, the percent drug content of liposomal AmB was found to be very low when compared to other solvent systems because of the low stability of AmB in the MeOH + CHCl_3_-HCl solvent system. The percent drug content of liposomal AmB using DMA-vit C was slightly higher than that using DMF-vit C. Finally, based on the above findings, the DMA-vit C system was chosen as an optimal solvent system for liposomal preparation. Furthermore, this solvent was used throughout this experiment.

#### 3.2.2. Effect of Temperature and Pressure on the SCF-CO_2_ Process 

Table 3 shows the mean diameter, PDI, and EE % of liposomal AmB produced by the SCF-CO_2_ process while using DMA as a solvent at various conditions of temperature and pressure. The range of temperatures was between 35–60 °C, whereas the pressure varied between 10–30 MPa. In all of the experiments, the dried lactose particles coated with the drug-lipid mixture were hydrated at 65 °C for 40 min.

It was observed that formulations that were prepared at 10 MPa pressure and 35 °C temperature did not represent appropriate conditions for the liposomal AmB preparation. Liposomes were formed at a pressure of 15 MPa; however, the EE % of liposomal AmB prepared at a constant temperature were decreased with increasing pressure. In addition, the percent EE of AmB was significantly decreased by increasing the temperature of the SCF-CO_2_ process by 45 °C.

Among the pressure and temperature conditions evaluated, liposomes that were prepared at SCF-CO_2_ process conditions of 15 MPa and 45 °C exhibited the best results in all of the parameters tested. The stable liposomes were formed and the size of the liposome, unlike the EE %, was not affected by the temperature and pressure of the process. Therefore, further experiments of liposomal AmB prepared by the SCF-CO_2_ process utilized a temperature of 45 °C and a pressure of 15 MPa.

#### 3.2.3. Effect of Lipid Concentration on the EE of Liposomes 

**Figure 4:**
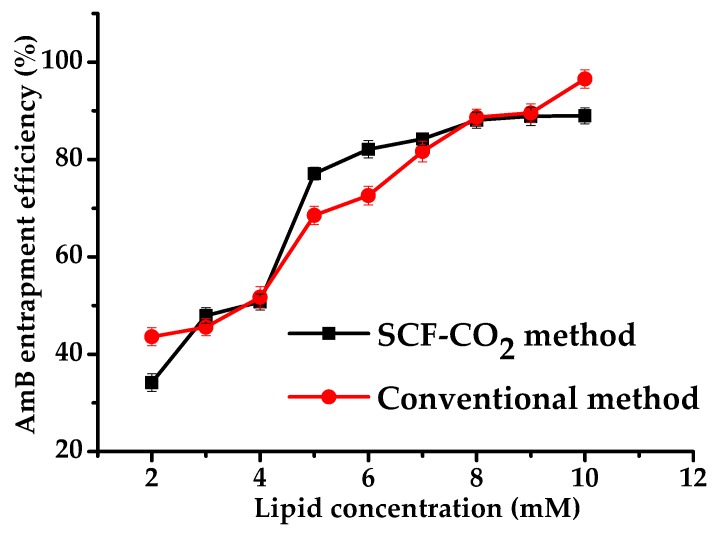
Encapsulation efficiency of liposomal AmB prepared by the SCF-CO_2_ and conventional methods.

Figure 4 shows the effect of lipid concentration on the EE of liposomal AmB prepared by the SCF-CO_2_ and conventional methods. The EE increased with increasing lipid concentration and was found to be similar in both methods (SCF-CO_2_ and conventional). This result was similar to the previous observations of increased EE % with increasing lipid concentrations for doxorubicin and protein loaded-liposomes [27,28], but different from that of the results of Otake et al., in which a water soluble compound, glucose, was used [29,30]. In the present study, both of the methods showed nearly 90% of EE at high lipid concentration, which revealed that a hydrophobic drug was more easily entrapped in liposomes than a water-soluble drug. Additionally, it was suggested that the EE of the hydrophobic drug was less affected by the size and lamellarity of liposomes than the water-soluble drug. However, the factor that had the greatest effect on EE in both methods was lipid concentration.

### 3.3. Lamellarity of Liposomes

**Figure 5:**
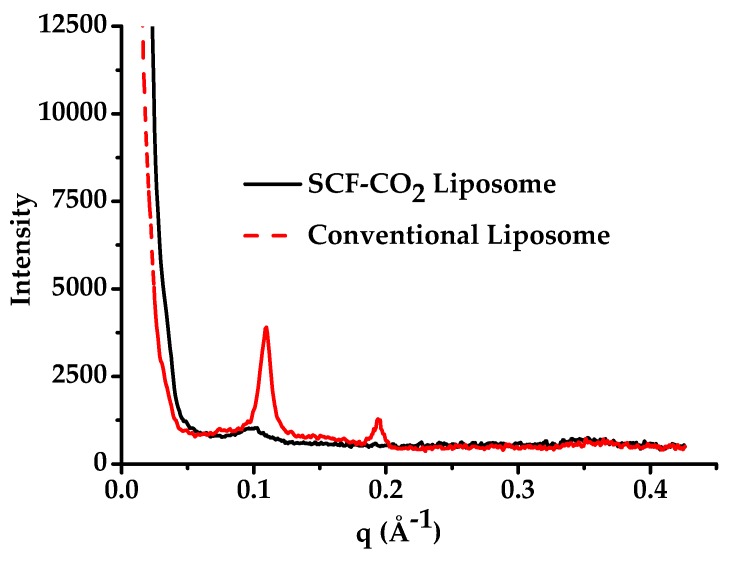
Small-angle X-ray scattering (SAXS) curves of liposomal AmB prepared by the SCF-CO_2_ and conventional method liposomes.

Liposomes are unilamellar or multilamellar vesicles; therefore, particle size varied with the number of lamellarity. The lamellarity of liposomes that were prepared in the present study was determined by small-angle X-ray scattering (SAXS). Figure 5 presents the SAXS profiles of the SCF-CO_2_ liposomes (black lines) and conventional liposomes (red dash lines), respectively. SAXS was used to obtain the global features of lamellarity of the samples, as TEM can only image the liposomes [31]. No sharp scattering peaks were observed for the SCF-CO_2_-processed-liposomes, whereas there were at least two peaks for the conventional liposomes. Moreover, for polydisperse systems, the absence of peaks for SCF-CO_2_ liposomes was caused by the superposition of many vesicle of different thickness and sizes, which demonstrated that there were no multilamellar vesicles after the micro-fluidization process of the liposomes [32].

### 3.4. Morphology of Liposomes

#### 3.4.1. Freeze-Fracture Electron Microscopy

**Figure 6:**
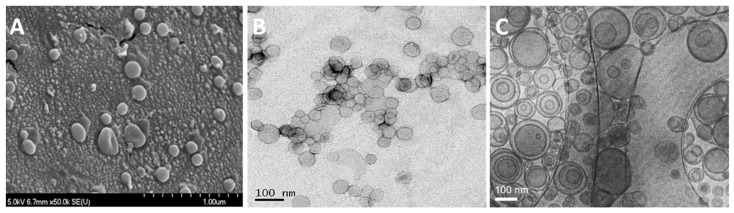
Morphology of the AmB-liposomes. (**A**) Freeze fracture electron micrographs; negative-stain transmission electron micrographs of liposomes prepared by the (**B**) SCF-CO_2_ method, (**C**) conventional method.

The morphology and structural characterization of the liposomes was carried out by the freeze fracture electron microscope. Figure 6A shows the freeze fracture electron micrographs of liposomes that were prepared by the SCF-CO_2_ method at 50× magnifications. These figures confirmed that most of the liposomes were spherical with a varying diameter of 0.1–1.0 μm. However, the majority of liposomes were less than 200 nm in size with fewer large-sized liposomes.

#### 3.4.2. Transmission Electron Microscopy

The liposomal AmB prepared by the SCF-CO_2_ and the conventional method was observed while using the negative-stain transmission electron microscope (TEM) to characterize the morphology of the liposome. Figure 6B,C show the transmission electron micrographs of the liposomes that were prepared by the SCF-CO_2_ and the conventional method, respectively, at a scale bar of 100 nm. In the case of the SCF-CO_2_ method, no multilamellar liposomes were observed, which suggests the presence of vesicles with non-concentric bilayers. However, conventional liposomes (Figure 6C) revealed at least two lipid bilayers confirmed the multivesicular structure of the liposomes. Moreover, SCF-CO_2_ -processed-liposomes reveal a uniform spherical shape (sized between 50–200 nm) with a smooth surface (Figure 6B).

### 3.5. Effect of Lyophilization on Liposomal AmB

Karn et al. [22] reported that the lactose used as the carrier in this study played an important role in cryoprotection. In this experiment, 9% lactose solution was prepared to evaluate the effect of lyophilization on liposomal AmB. Table 4 shows the size, polydispersity, zeta potential, yield, and EE % of the liposomal AmB before and after the lyophilization. No significant change was observed in any of the parameters tested.

### 3.6. Stability of Liposomal AmB 

**Figure 7:**
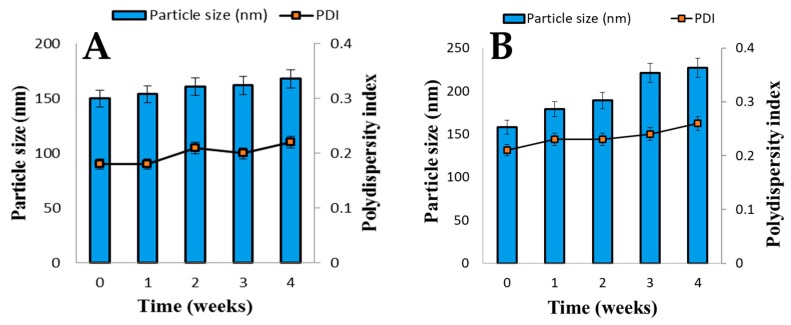
Change of particle size and polydispersity index (PDI) of (**A**) SCF-CO_2_ liposomes, and (**B**) conventional liposomes stored at −5 °C over time. Data are expressed as the mean standard deviation (*n* = 4).

Liposomes were found to be the most stable at a storage temperature of −5 °C. SCF-CO_2_ and conventional liposomes were both stored at −5 °C, and Figure 7A,B, respectively, show the results of the change of particle size and PDI of SCF-CO_2_ liposomes and conventional liposomes. The conventional liposomes were aggregated just after one week of storage, whereas no significant changes were observed in the particle size of the SCF-CO_2_ liposomes during four weeks of storage, which indicates that there was no aggregation.

### 3.7. Hemolysis Properties

**Figure 8:**
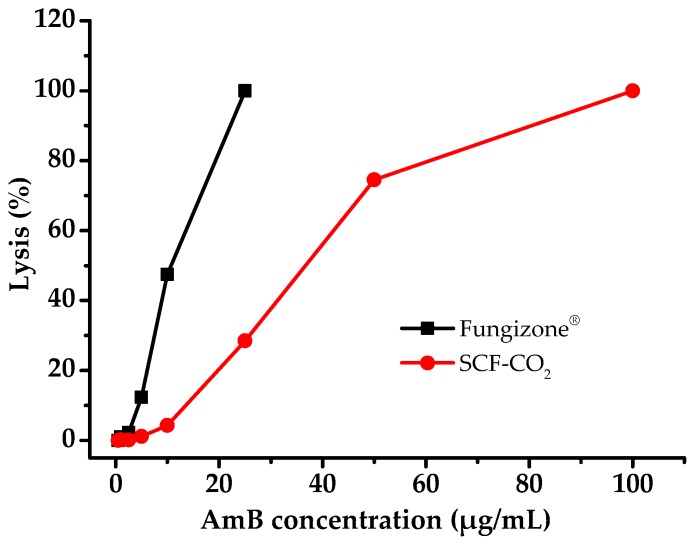
Hemolysis of rat RBCs at various concentrations of AmB as Fungizone^®^ and liposomal AmB prepared by the SCF-CO_2_ process.

Intravenous administration of AmB reported that interaction with RBC causes anemia. Thus, the safety of liposomal AmB can be studied by comparing with the micellar Fungizone^®^ for their hemolysis potential. The result of the hemolytic test of liposomal AmB was compared with that of Fungizone^®^, as shown in Figure 8. The liposomal AmB that was prepared by the SCF-CO_2_ process was slightly affected, up to 10 μg/mL of AmB concentration, whereas a micellar formulation Fungizone^®^ showed almost 50% hemolysis at the same concentration of AmB.

The reason behind this could be that the lipid bilayers of liposomes reduce direct contact of AmB with erythrocytes. Therefore, it was considered that liposomal AmB that was prepared by the SCF-CO_2_ process was less hematotoxic than a micellar formulation of AmB, Fungizone^®^.

### 3.8. In Vivo Pharmacokinetic Study

**Figure 9:**
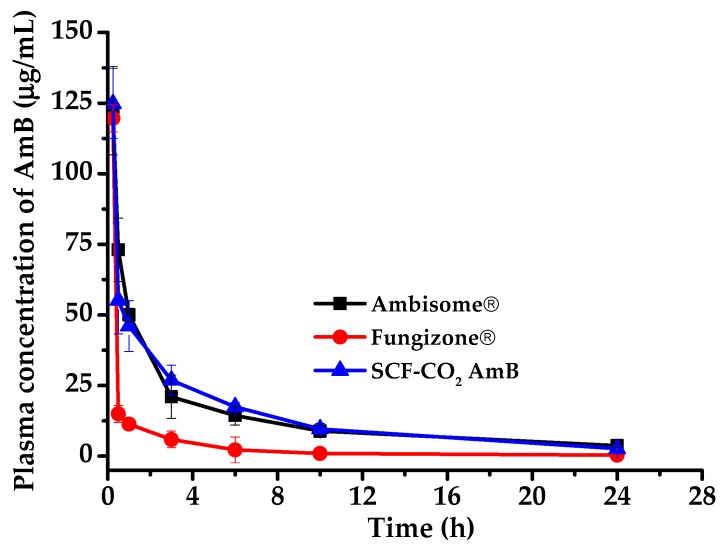
Plasma concentration-time profile of commercial products (AmBisome^®^ and Fungizone^®^) and the SCF liposomal AmB after administration as a single dose of 3 mg/kg by a tail vein injection. Data are expressed as the mean standard deviation (*n* = 4).

In this study, SCF-CO_2_-processed-liposomal AmB observed smaller particle size with uniform distribution and higher EE. Therefore, the pharmacokinetic evaluation of the SCF-CO_2_-processed-liposomes was of the utmost necessity. SCF-CO_2_-processed-liposomal AmB was evaluated for pharmacokinetic parameters in rats. AmB commercial products (AmBisome^®^ and Fungizone^®^) and SCF liposomal AmB were administrated as a single dose of 3 mg/kg [33,34] by tail vein injection. Figure 9 shows the mean plasma concentration–time profiles for the formulations evaluated Group 1: AmBisome^®^, Group 2: SCF-CO_2_ liposomal AmB, and Group 3: Fungizone^®^. Through the lateral-tail vein, the time to reach maximum plasma concentration (T_max_) was observed at 0.25 h (i.e., the first sampling point); however, 0.25 h was the time that was taken to reach peak plasma concentration (C_(0.25 h)_). Therefore, the relevant pharmacokinetic parameters, including C_(0.25 h)_ (peak concentration at 0.25 h), the area under the plasma level-time curve (AUC_0–24 h_), which was obtained by the trapezoidal rule, and the AUC_24 h–∞_ time, which was determined by dividing the last plasma concentration by k_e_ and adding this result to the AUC_0–24 h_, are listed in Table 5. The plasma concentration–time profile of liposomal formulations (AmBisome^®^ and SCF liposomal AmB) and colloidal dispersion (Fungizone^®^) were quite different. It was observed that liposomal formulations showed similar C_(0.25 h)_ as compared to that of the colloidal dispersion Fungizone^®^. C_(0.25 h)_ of 122.28 ± 15.60, 124.83 ± 12.41, and 119.61 ± 0.76 µg/mL were observed for AmBisome^®^, SCF liposomal AmB, and Fungizone^®^, respectively. Interestingly, the overall AUC_(0–24 h)_ for SCF liposomal AmB was observed to be similar to that of the commercial liposomal formulation AmBisome^®^; the values are 316.79 ± 60.46 and 325.64 ± 32.76 µg∙hr/mL, respectively. AUC_(0–24 h)_ was observed to be more than 4-fold lower for Fungizone^®^, which demonstrates the superiority of our liposomal formulation.

## 4. Discussion

### 4.1. Screening of Organic Solvent Systems

AmB was very unstable in organic solvents with HCl, whereas it was considerably stable in organic solvents with vit C. The reason for this higher stability was that vit C protects AmB from autoxidative degradation due to its antioxidant properties [35]. The current study suggested that vit C appeared to be an effective stabilizing agent for AmB in organic solvents. This was probably because the AmB solubility in acidic conditions increases as a result of decreasing intramolecular interactions [36]. 

The organic solvent was mixed with SCF and the vit C was rapidly precipitated from the organic solvent when the SCF was introduced into a vessel containing an organic solvent with vit C. As a result, the solubility of AmB in DMA and DMF was considerably decreased in supercritical conditions and most of the drug remained on the surface of lactose. In contrast, DMSO maintained the high solubility of AmB in the supercritical condition and a portion of the drug was drained out at the washing step.

### 4.2. Optimization of the Preparation Process for Liposomal AmB

The success of preparation of liposomes while using the SCF-CO_2_ method depends on the solubility of AmB and lipids in the SCF-CO_2_. However, visual observations revealed the very low solubility of AmB and lipids in the SCF-CO_2_ at pressures of up to 20 MPa. The insoluble AmB could not be entrapped in liposomes; therefore, the complete dissolution of AmB in SCF-CO_2_ was needed. The organic solvent is an inhibitor of liposome formation and an excessive amount destroys the liposomes. Therefore, a minimum volume of organic solvents was used throughout the experiment.

The principle of the SCF-CO_2_ process that was used in this study is similar to that of the antisolvent properties of the SCF-CO_2_, in which the success of the SAS process depends on the solubility of the liquid solvent in the SAS and the fact that the solute is not soluble in the antisolvent [20,37]. It is necessary to find the optimum temperature and pressure conditions for the selective precipitation of the solute from its solution.

Pressures that were below 10 MPa and temperatures below 35 °C were not suitable for the preparation of liposomal AmB due to the residual organic solvent in the reaction vessel; this might be attributed to the subcritical state of the mixtures of CO_2_ and organic solvents. The diffusion coefficients at subcritical temperatures were smaller than those at supercritical temperatures [38], resulting in the inability to remove the organic solvents. The drug percent content of liposomal AmB was decreased with the increasing temperature of the SCF-CO_2_ process; this can be explained by the increased solubility of the drug in supercritical carbon dioxide at high temperatures, which led to an increase in the density and dielectric constant of the supercritical carbon dioxide, resulting in the higher solubility of the drug in the supercritical fluid [20]. In this study, the liposomal AmB that was prepared by the SCF-CO_2_ process at 45 °C, 15 MPa showed the highest yield and EE% along with the smallest size. Therefore, this condition was considered as the optimized condition for liposome preparation using the SCF-CO_2_ method.

Based on SAXS scattering peaks in Figure 5, it could be concluded that liposomal AmB that was prepared by conventional methods has a multivesicular structure. In the case of the SCF-CO_2_ method, no diffraction peak was observed, which suggested the presence of vesicles with non-concentric bilayers or a single bilayer. Transmission electron micrographs of the liposomes revealed unilamellar morphology of the SCF-CO_2_-processed-liposomes (Figure 6B). However, conventional liposomes (Figure 6C) revealed at least two lipid bilayers confirmed the multivesicular structure of the liposomes. The lyophilization of liposomes is one of the best ways to circumvent many of the stability problems associated with liquid liposome suspensions; thus, this is a frequently used technique to improve the stability of systems that are unstable in an aqueous environment, such as proteins and liposomes [19,27]. It has been reported that the liposomes containing drug molecules can be lyophilized and reconstituted with significant drug retention, which is measured as the percent of the encapsulated drug and without a significant change in the mean vesicle size. Cryoprotectants are needed during lyophilization, and sugars, such as sucrose, lactose, and trehalose, are used to protect the liposomes during the freezing stage of the lyophilization cycle [39,40]. Lyophilization with 9% lactose as a cryoprotectant provided physical stability to the liposomes as well as high protection against liposomal drug leakage. Therefore, the lactose used in the preparation of these liposomes not only acted as the carrier particle, but also, more importantly, confirmed the cryoprotectant activity. The stability of liposomes that were prepared by the SCF-CO_2_ might be explained by the static repulsion of the carbonic acids incorporated into the bilayer membrane; however, the lyophilization process would be recommended for the long-term stability of liposomes [30,41,42]. The high hemolytic behavior of Fungizone^®^ indicated that the release of AmB from a micellar formulation was faster than that of a liposomal formulation of AmB [43]. In addition, this result supported the fact that the lipid bilayers of liposomes can lower the direct contact of AmB with erythrocytes and the release of AmB from liposomes due to the interaction between AmB and phospholipids and/or cholesterol [44]. Therefore, it was considered that liposomal AmB that was prepared by the SCF-CO_2_ process was less hematotoxic than a micellar formulation of AmB, Fungizone^®^.

## 5. Conclusions

In this study, we developed liposomal AmB that was prepared by the SCF-CO_2_ method. Firstly, various solvent systems were investigated; subsequently, the DMA-vit C system exhibited high solubility and stability of AmB. In addition, the EE % of liposomal AmB while using the DMA-vit C system was higher than that of any other solvent systems. This solvent system was used to optimize the process conditions. The liposomal AmB that was prepared by the SCF-CO_2_ process at 45 °C, 15 MPa showed the highest yield and EE % and the smallest size. The percentages of entrapment of AmB in the SCF-CO_2_ liposome formulations were more than 90%. The microfluidization of liposomes resulted in uniformly distributed spherical particles, which were approximately 100~150 nm in size. No significant changes in particle size, EE, and PDI of liposomal AmB before and after the lyophilization were observed. Transmission electron micrographs of the liposomes revealed unilamellar morphology of the SCF-CO_2_-processed-liposomes; however, at least two lipid bilayers for the conventional liposomes. The SCF-CO_2_-processed-AmB formulation exhibited higher stability than the conventional liposomal formulation. The in vivo pharmacokinetic study results demonstrated that liposomal AmB that was prepared by the SCF-CO_2_ process was less hematotoxic than a micellar formulation of AmB, Fungizone^®^.

The SCF-CO_2_ that was described in this study was one of the simplest and advanced methods and it produced non-toxic liposomes. Therefore, this study was successful at not only identifying the effective parameters in formulating liposomal AmB using the SCF-CO_2_ method, but also determining its advantages in the treatment of antifungal infections, as compared to other conventional formulations of AmB.

## Figures and Tables

**Table 1:** The pseudo-first-order rate constants (*k_obs_*) and half-life (*t*_1/2_) values of AmB in different organic solvent systems.

Organic Solvent Systems	*k*_obs_ (hr^−1^)	*t*_1/2_ (h)	R^2^
DMSO	0.1244	5.57	0.888
DMSO-vit C	0.0594	11.66	0.746
DMSO-HCl	5.5074	0.13	0.874
DMA-vit C	0.2808	2.47	0.986
DMA-HCl	2.4844	0.28	0.97
DMF-vit C	0.2949	2.35	0.891
DMF-HCl	15.5025	0.04	0.981
MeOH + CHCl_3_-vit C	Not Done	Not Done	Not Done
MeOH + CHCl_3_-HCl	2.2989	0.30	0.953

**Table 2:** Effect of organic solvent on liposomal AmB prepared by the supercritical fluid of carbon dioxide (SCF-CO_2_) method.

Organic Solvents	Size (nm)	PDI	EE (%)
DMSO	771 ± 89	0.25 ± 0.01	81.2 ± 2.2
DMSO-vit C	833 ± 78	0.22 ± 0.02	82.0 ± 1.9
DMA-vit C	849 ± 108	0.21 ± 0.02	91.5 ± 2.3
DMF-vit C	1109 ± 112	0.21 ± 0.01	90.2 ± 3.0
MeOH + CHCl_3_-HCl	692 ± 62	0.19 ± 0.01	35.8 ± 5.2

**Table 3:** Effects of pressure and temperature on the SCF-CO_2_ process of liposomal AmB.

Temp. (°C)	Pressure (MPa)	Mean Diameter (nm)	PDI ^a^	EE (%) ^b^
35	20	N/A	N/A	N/A
45	10	N/A	N/A	N/A
45	15	761.0 ± 56.5	0.19 ± 0.01	91.7 ± 4.2
45	20	949.3 ± 84.4	0.23 ± 0.01	90.5 ± 5.7
45	25	855.7 ± 76.2	0.25 ± 0.02	84.2 ± 2.9
45	30	821.3 ± 69.2	0.27 ± 0.01	79.7 ± 5.5
55	20	801.6 ± 74.9	0.20 ± 0.02	86.3 ± 4.5
65	20	839.1 ± 81.7	0.18 ± 0.01	77.1 ± 4.2

Note: Values denote the mean ± standard deviations of four separate sets of experiments. N/A denotes not applicable, i.e., liposomes were not formed. ^a^ Polydispersity index, ^b^ Encapsulation efficiency.

**Table 4:** Influence of the lyophilization process on particle size, zeta potential, yield, and encapsulation efficiency of the SCF-CO_2_.

Condition	Size (nm)	ZP (mV)	Yield (%)	EE (%)
Before LP	137.3 ± 7.3	−42.5 ± 1.0	91.6 ± 1.2	89.2 ± 1.8
After LP	146.8 ± 2.2	−43.6 ± 1.7	90.2 ± 1.0	88.9 ± 2.2

LP, lyophilization; ZP, Zeta Potential; EE, Encapsulation efficiency.

**Table 5:** Comparative in vivo pharmacokinetics parameters observed in rats (*n* = 4).

Parameters	Groups		
	AmBisome^®^	SCF-CO_2_ Liposomal AmB	Fungizone^®^
C_(0.25 h)_ (µg/mL)	122.28 ± 15.60	124.83 ± 12.41	119.61 ± 0.76
AUC_(0–24 h)_ (µg·h/mL)	316.79 ± 60.46	325.64 ± 32.76	76.10 ± 1.56
AUC_(0–∞)_ (µg·h/mL)	369.45 ± 60.06	349.81 ± 28.50	79.66 ± 2.74
*t*_1/2_ (h)	9.76 ± 1.74	6.25 ± 0.50	6.98 ± 1.50

SCF liposomal AmB vs. Fungizone^®^ *p* = 0.0343; AmBisome^®^ vs. Fungizone^®^ *p* = 0.0406; SCF liposomal AmB vs. AmBisome^®^ *p* = 0.8924. *p* < 0.05 represents a significant difference by two-sided RM ANOVA and Bonferroni test.
